# Better Babies*Address given at the Better Babies Contest, San Antonio, November 8, 1913.

**Published:** 1914-04

**Authors:** Theo. Y. Hull

**Affiliations:** San Antonio, Texas


### Department of Eugenics.

Conducted by Dr. Malone Duggan and Dr. Theo. Y. Hull.

### THE TEXAS STATE SOCIETY OF SOCIAL HYGIENE.

Headquarters: San Antonio, Texas.

Dr. Malone Duggan, President, San Antonio.
Dr. F. E. Daniel, 1st Vice President, Austin.
Prof. A. Caswell Ellis, 2d Vice President, Austin.
Mrs. Eli Hertzberg, 3d Vice President, San Antonio.
Dr. Theo. Y. Hull, Secretary, San Antonio.
Mr. W. L. Evans, Treasurer, San Antonio.

EXECUTIVE COMMITTEE.

Dr. Malone Duggan, Chm.
Dr. Theo. Y. Hull, Sec'y.
Dr. W. F. McCaleb.
Hon. C. H. Jenkins.
Rev. A. W. S. Garden.
Hon. J. C. Willacy.
Mr. W. L. Evans.
Dr. Frank D. Boyd.
Dr. J. R. Nichols.
Dr. J. M. McCutchan.
Dr. W. A. King.
Dr. J. W. Torbett.
Dr. F. C. Walsh.
Dr. Joe Reuss.
Dr. Frank Paschal.

### Better Babies.*

BY DR. THEO. Y. HULL, SAN ANTONIO, TEXAS.

I am glad of the opportunity to say even a few words in behalf of that greatest of all institutions, the American baby. I consulted the Century Dictionary for a concrete definition of the term "baby." It is said to be both a noun and an adjective. I had come to believe it an interjection or an exclamation point. We might describe the baby as a bundle of unlimited possibilities, both active and passive, characterized by a vast difference between

*Address given at the Better Babies Contest, San Antonio, November 8, 1913.

the expectation and the realization. The better baby is an attempt to equalize the two terms of the equation.

A few months ago when we were discussing the Owen bill, then pending before Congress, in this room Mrs. Laura B. Hart spoke of the American baby as "our most important national asset." Yet of all American products the baby has been the most neglected, not only by the nation but the State, which never seems to realize that it has any rights unless they are in some way associated with other tangible assets. The only law of inheritance the government recognizes is that of property.

There are so many possibilities tied up in the baby. Most of us were babies. Some of us now realize how many of these possibilities our parents got out of us—and those largely expressed in kicks and yells of protest at some imaginary wrong.

When you come to think of this subject, "Better Babies," what a vista it opens up, what a breadth, what a depth. The vista leads all the way back to Adam and Eve, who should have been better instructed in the beginning. Had they been better instructed we would not have so many of what the Century Dictionary calls blue babies. They would all have been round, plump, equally developed, loveable babies. In breadth there is nothing wider. It reaches all around the world. Better babies are desired in every nook and corner—that is, babies that give less trouble. In depth, the subject reaches down to the very bottom of the wells of human nature.

This better baby is a product of three factors, good parents, good food, and good surroundings. Each one contributes its share. Omit one and the product is spoiled. Of these factors the first is the most important. The second and third contribute the opportunity for developing the good, the great possibilities tied up in the baby.

An eminent physician when called to see an epileptic child told the mother that a consultation should have been held. She replied that one had been held. "Yes, madam," he answered, "it should have been fifty years ago."

Now I am going to tell you some of the things that make bad babies, and by bad babies I mean sickly, undeveloped, deformed, those upon whom some blight has fallen. Because of no fault of theirs, they are doomed to a life, sometimes short, somehow long, but always below and behind the normal.

Back of the child is the parent, the grandparent and a long line of ancestors, some good and some bad. The baby is what those permit it to be. They made the possibilities. It could be no more. We want better mothers for these better babies, and we might have better fathers also.

Did you ever study a feeble-minded child, an imbecile, an idiot? What made it so? It is not sick. It is just as well as any other child. But somehow and for some reason its mental development stopped and no medicine, no power can in any degree correct the condition. The diseased may be cured. The deformed may sometimes be made straight, and even the blind may in a few instances be made to see, but for the feeble-minded there is no hope. If it lives it must go through life with no intellect or an intellect anywhere below that of a thirteen-year-old child. It may become a public charge unless kind Providence exempts it from further misery or it may continue to eke out a bare existence under the simplest conditions. It has no place in our complex system of living and competition. It gravitates to the lower strata where sorrow, disease and crime reign almost unmolested. What made it thus? Who is to blame? The parents have reveled in vice, in disease, it may be in crime and because of their guilt either permitted or premeditated the offspring is doomed to this. And society, to which we belong, laughs at the Simple Simon of our streets, hands out a scornful bit for the beggar and teases the garulous old woman who sits in the corner when it should pity. These derelicts have not sinned but their parents before them. We see those things and take no steps to prevent them

Did you attend the funeral of your neighbor's baby this morning? Do you know that there has been nearly 1000 such funerals in this country today? Do you know that every fourth death notice you read is that of a child under two years old? Do you know the number that are born dead, that live one day, one month, one year? Do you realize the waste this implies, I might with equal propriety say crime this implies. Approximately 320,000 children die each year under two years of age. This number is nearly 25 per cent of all the deaths in the country. This is a large number. Yet 260,000 of those died during the first year. Nearly 40 per cent died under one month, and 10 per cent were infants who died before the completion of the first day of life. I don't like to use such figures. They are too big, too terrible, when you attempt to translate them into terms of human misery, sorrow, degradation and crime, for some of it is crime. To think of all those homes laid waste, a quarter of a million each year, benumbs our senses. We are not able to comprehend them.

It is said that nearly 70 per cent of this loss is preventable. This certainly concerns us in our study of why not better babies.

The cause is important. Of every 100 of these dead infants, 29 died of diarrhea, 13 of premature birth, 10 of congenital debility and 5 of malformations. This has a very grave significance. It means that of those born alive these did not have sufficient vitality to endure the first trials of life. They came into existence so frail that the first contrary wind carried them away. Diarrhoeal troubles mean poor food, either natural or artificial, nearly always the latter. Malformation and congenital debility mean usually that one or both parents were sick. Often one or two chronic maladies are responsible for the failure of vitality and development. Premature birth may mean active parental disease or interference, very largely the former. It is highly probable that over 50 per cent of the babies that die under one year, die because their parents, one or both, were in a state of health which did not permit of healthy offspring and were therefore unfit for parenthood.

Parenthood is a necessity and yet it entails a grave responsibility, for unfit offspring are rarely other than a sorrow, often a distinct curse. The mentally and morally deficient must expect mentally and morally deficient offspring. Those suffering from certain active and chronic diseases must not expect their offspring to be perfect in form and physically well. It is an old saying and a true one that like begets like, a chip off of the old block.

There is still another class of parents we ought to consider. I sometimes think of them as starved parents. These are the parents that live in tenements and toil in sweat shops, sometimes designated as factories of human woe. Did you ever stop to think that a large per cent of these human machines—for literally, machines they are—rarely know what it is to have a bountiful meal as you and I know it? Yet these people live and beget their kind in superabundance to suffer a similar starved existence. Misery loves company. In one of our States there are over 30,000 men and women, human oxen, toiling and starving for a pittance of less than $4.50 a week. In another State there are over 300,000 industrial workers—unskilled labor it is usually called—who draw less than $7.50 per week on which to support a home and rear a family, and these families range from three to eight members.

What is the result of this condition? It is simply this: The mothers must also labor in the factory to increase the family income to the living point. What does this mean? If you will study the vital statistics of those communities in which this condition prevails, you will see for once the relation of cause and effect. It is a matter of general comment that in cities and communities where the women are industrially employed there is a high rate of infant mortality and indeed a high rate of mortality for all ages, and it is assumed that the high infant death rate, those under one year, is due to the employment of the mothers.

In one American city where a large per cent of the married women are so employed, for every 100 deaths, 38.4 were those of children under one year, exactly double the rate for the entire country. As a sort of compensatory evil the birth rate was exceedingly high—43 per 1000 population. To follow out the comparison with the general death rate of each 100 infants dying under one year, 38.3 died of diarrhea.

This increase in the death rate was not due to the mere fact that the mothers of a part of them worked in mills or factories, for all the mothers of this class work, and work hard, but it was due to hard work coupled with insufficient food and unhygienic surroundings for the expectant mothers, which resulted in weakened offspring. When to this an increase in artificial feeding is added the increased death rate is readily accounted for.

The influence of these conditions and other conditions which interfere with the nutrition of the expectant mother upon the offspring must be considered. Many of these mothers because of the character of the food which they are compelled to consume suffer from digestive disorders which brings about a starved condition of the baby. Excessive toil likewise affects the nutrition of the mothers. These conditions are imparted to the embryo, which fails in development. The child comes into existence a weakling, which the first struggle with improper food or infantile diseases terminates in death.

Over-worked mothers and poorly nourished mothers are therefore unfit mothers. There is another class of prospective mothers that receive very little consideration. These are to be found in every city and in every community. Shop girls, we sometimes designate them. Every one of them expects to marry some time, but many of them never marry. The conditions under which they labor are such as to jeopardize their health. Long hours and continuous nervous strain wear out or derange the frailer members. It was an unhappy day when woman was compelled to enter into industrial compaction with man. Nature intended woman for a higher sphere. It endowed her with high intelligence, with finer sensibilities and greater charms than man not to adorn a work shop or a bargain counter, but to be the center of and grace a home. The ideal woman is neither the toiling woman, nor the professional woman. It is the mother, and when by toil or by other unfavorable conditions she fails in this she fails in the one great purpose of her being.

On woman falls the burden of the perpetuation of the race. Better babies must come from better mothers. Neither the mentally deficient, the physically weak, the diseased, nor the overworked and underfed nor those that labor under unnatural conditions are fitted for the highest of all duties, motherhood. Medicine cannot make these fit. Fitness must come with health, physical, mental and moral worth.

The aim of science, of sanitation, of hygiene of today is to save life, to prolong life and make life worth the living. Long ago Huxley declared that the chief end of human evolution was not so much the survival of the fittest as the fitting of as many as possible to survive. It has been said that the care and training of the baby should begin a century or two before its birth. Only normal parents can beget and give birth to normal children. The birthright of every child born into the world is a clear brain and a strong body, and this can only be insured by a clear brain and a strong body in the parents. The first factor, the good parents, is the strongest factor in the better baby.

The second strong factor in the equation of the better baby is proper and sufficient food. Whenever you depart from this either in quality or quantity the organism fails. The fact that so many infants die of diarrheal troubles, especially during the earlier months, signifies poor food. The fact that so many of those who do survive are thin and pale and starved in appearance means insufficient food. The additional fact that so many of those infants whose life flame was snapped out ere it began to glow were fed, not nourished, on artificial food, means that that many mothers did not or could not nourish their babies as nature intended. There are three classes of mothers. The one who has few children or only one and has no time to nurse it because it is not convenient. The opposite class has many children and cannot nurse them, no matter how strong the maternal instinct, because of poverty. In the first case the baby loses its birthright to be nursed at its mother's breast because it is not convenient. In the other class it loses its birthright because it is impossible. To this class some day the government will learn its duty to insure a living wage. The third class, the great middle class, the bone and sinew and brains of the nation, recognize the birth of the child and the mother instinct and fail not their duty.

The effects both of improper food and insufficient food are most apparent in the earlier months. In this period improper food is apt to terminate the infant's existence while insufficient food may check its development. Practical experiments show that animals stunted by an insufficient supply of food after a time lose their ability to develop when a sufficient normal diet is supplied. Observation upon babies show that the young organism will overcome short periods of restrained growth, but when the low diet is continued too long, as it sometimes does over the entire period of youth, the child loses its ability to develop proportionately. The younger the organism when the restriction of diet occurs the greater the injury.

When we consider the chemical composition of the human body we realize that the food must contain certain things, and if it does not contain them the body suffers most during early life. When lime and phosphorous are withheld below a certain limit the bones do not develop or we have rickets. Milk is an ideal food for the infant, but milk is poor in iron, and if it is continued too long the blood suffers a diminution in its hemoglobin and the child suffers from anemia. Water is a very important constituent of the food. Young animals drink water freely. Experiments on two young dogs—in which each were given the same amount of food—the one with a limited supply of water and the other with water, freely showed that the one with all the water it desired outgrew the other. We may know that poor food means poor development. This has been proven by experiments and observation on plants and animals. The children of the poorer classes in every country are of lighter weight and smaller size than those of the socially better off. Food may not be and probably is not the sole factor in this difference between the children of the poor and the children of the well to do, but it is an important factor. When this factor of poor food is grafted on the more important factor of bad inheritance we can foretell with certainty poor development.

The third factor in this better baby is that of its surroundings—sunlight, quiet and fresh air—are first essentials. Sunlight is essential to plant life, for in it the processes of life go on freely. Plants grow tall in an effort to reach the light. Light is necessary to child life also. Dark apartments and gloomy surroundings depress the vitality. The natural instincts of young animals lead them into the open. You rarely see them hiding away in the dark. If they do so it is from fear. The baby is a young animal, and it will be the better baby for being in the open. Fresh air is just as essential as food. The child needs oxygen to make use of the food it has eaten. This must come from the air. The purer and more abundant the supply the more rapidly will the young organism develop. How many houses are so built as to provide a free circulation of air? How many mothers close every avenue for its free circulation? For how many hours is the child compelled to breathe over and over the same air? No mother would think of feeding her baby on spoiled milk or give it a drink of water from a stagnant pool. Yet many are the mothers who will do practically this very thing in regard to air. Food is taken at intervals of several hours. Air is taken by the baby at intervals of less than three seconds during the entire twenty-four hours. There is another thing the baby needs and not infrequently fails to get. It is rest. The baby is not a toy, a plaything. It is a young human being. In its earlier months sleep is its greatest need. When it fails to get rest its sensitive nervous system pays the penalty, and every organ in the body feels the depression. Later it grows up demanding a constant surfeiting of excitement, an unhealthy mental condition indicating a want of stability. The better baby of the better mothers will have rest in which its delicate organs adjust the tiny being to its surroundings.

In conclusion I wish to impress upon you that the first essential for the better baby is to be better born, well born. To accomplish this we must teach and treat the parents, and better still, the grandparents. Take marriage out of the realms of convenience, love at first sight and affinity. Inject a large dose of common sense into human matings. Bar the sick, the feeble-minded and the morally perverted. Since the second essential to a healthy normal body is to be well fed, let both the prospective and the expectant mother realize her high calling and prepare to do her duty. It is simply the right of the unborn that this should be done. Relegate the poodle dog and the thousand and one things that take the mother's attention from her baby to the realm of the spinster. She must have something to do. And lastly, realize that the human baby is a young animal of the highest type, that the things that injure the young of other species, injure it also, only to a greater degree. Make its environment what it should be. The most perfect objects in nature are found where the conditions are best. Surround this, the highest of earthly creatures, with the best conditions for physical and mental growth.

---

In differentiating syphilitic from other bone lesions a negative Wassermann reaction is not diagnostic.—*American Journal of Surgery.*

